# On humans and their crops—miRNAs and the evolution of fertility

**DOI:** 10.1007/s00709-020-01595-w

**Published:** 2020-12-17

**Authors:** Peter Nick

**Affiliations:** Botanical Institute, Karlsruhe Institute of Technology, Karlsruhe, Germany

“*Natura non facit saltús*” (Linnaeus 1751)—“Nature does not make leaps” has been the guideline for causal analysis since the era of Greek philosophy. Even Darwin, although demonstrating that species are not constant, but changeable, conceptualized this change as gradual, proceeding in small steps, not in leaps. However, the more we learn about evolution, the more examples for rapid leaps accumulate. This includes our own species, as well as the species that we have shaped for our needs—domestication is a two-edged sword cutting both sides. Very small genetics can sometimes bring very much of evolution. How can we explain this? Obviously, evolutionary innovation cannot be a matter of revolutionarily new building blocks, but must result from new combinations of pre-existing elements. Innovation, thus, stems from new cross-connections between modules that are already in place. The discovery of microRNAs (miRNAs) as tool of cross talk has attracted, therefore, considerable interest, but its functional relevance is still not fully resolved. These single-stranded RNA species consist of usually 22 base pairs and do not encode proteins. However, they interfere with transcripts of coding genes, leading to their degradation or blocking their translation. After their discovery in the nematode *Caenorhabditis elegans*, it became soon clear that they exist in all eukaryotic life forms. Since they can originate from introns, they represent an important element of gene-gene communication. Expression of one particular gene will modulate the expression of a second gene, a phenomenon that genetically becomes manifest as epistasis. However, miRNAs can also derive from intergenic regions and it is type II or III polymerases (depending on the life form) driving their transcription. The loci encoding such miRNAs can expand even during short evolutionary periods, which will allow for massive changes of gene regulation. This might be one of the molecular mechanisms behind evolutionary leaps. Two contributions to the current issue address the functional consequences of such miRNA expansions. There is a curious coincidence—one of these case studies concerns *Homo sapiens*, the other *Triticum sativum*, a species that has arisen through the activity of *Homo sapiens*. This event launched a common history of domestication that has shaped both species substantially.

The contribution by Bullerdieck (2020) in the current issue deals with C19MC, a microRNA cluster on human chromosome 19. The precursor of this cluster is present in all mammals, but C19MC is specific to primates and evolved in a short period. The expression of this cluster is limited mainly to the embryo and the placenta. Thus, C19MC might have been crucial for human evolution. However, the function of this cluster has remained elusive. The author asked the question, whether expression of this cluster might be relevant for the functionality of the placenta, and therefore compared samples from spontaneous or induced abortions, along with matured placentas. They observed a considerable variation between different individuals, which was not manifested in serum samples. To understand the reason for these variations, they checked a possible correlation with the sample position within the placenta, but did not detect any. Furthermore, the expression levels seen in spontaneous abortions were not significantly different from those for induced abortions. The straightforward idea would have been that the expression of this cluster decides about continuation of gestation, but it is clear now that this idea does not hold. Although this is a negative result in the first place, it allows concluding that C19MC does not convey a housekeeping function. The individual variation in the expression of these miRNAs might reflect a role of these regulators for later (and primate-specific) stages of prenatal life.

The Mediterranean civilisations have been sparked by the domestication of wheat (*Triticum*) in the Fertile Crescent, around 8000 years ago. Two events of hybridisation followed by genome duplications (so-called allopolyploidy) gave rise to the hexaploid modern wheat able to feed large populations and, thus, enabling the formation of cities and states. Interestingly, this event is mirrored on the level of miRNA as addressed by the work by Yu et al. (2020) in the current issue. The microRNA family miR396, present in all Angiosperms, is conspicuously amplified in wheat with 17 members, some of which are unique and not found in other grasses. Using a combination of bioinformatics and expression analysis, the authors explore possible functions of this family. They show that the majority of the target genes belong to a group of plant-specific transcription factors involved in growth regulation. Of special interest are members involved with grain filling. The allopolyploidic events in the genesis of wheat did expand not only the miR396 family, but also their target genes, such that the regulatory network became enriched. This enriched regulatory network allows for a more intense partitioning of assimilates from the vegetative organs into the developing seeds. While wild grasses usually retain a part of their resources for vegetative development (often linked with a perennial life style), domestication led to a more efficient seed filling and, thus, to the active “decision” for an annual lifestyle. This would impair survival in the wild, but is beneficial for humans. It is possible, therefore, that we may understand domestication as a shift in regulatory paradigms.

In a much debated review, Mattick (2004) explains that with progressive evolution of multicellular organisms, the number of genes did not increase concomitantly with the complexity, while the non-coding parts of the genome proliferated. Using paradigms deriving from kybernetics, he further demonstrates that in a system consisting of nodes (genes) and lines (interactions between genes), the number of lines will increase with the square of the number of nodes. In other words, the regulatory complexity is increasing much faster than the complexity of the molecular players. Innovation comes from new interactions, not from new players. Non-coding RNA is a central tool to convey interactions between genes and, thus, should be seen as driver of evolutionary novelty. This driver can act rapidly, because the well-established nodes do not need to change. What changes, however, is their context. Thus, the full potential of the two contributions described above is unfolded only, when they are seen in this evolutionary context.
